# A Comparison of the Effect of Two Doses of Oral Melatonin as Premedication on Orientation Score, Induction Compliance, and Emergency Agitation of Children Undergoing Elective Surgeries: A Double-Blinded Randomized Trial

**DOI:** 10.1155/anrp/8832216

**Published:** 2025-03-14

**Authors:** Haider Muhy Al Bareh, Mohammed Jawad Kadhim Al Kidsawi, Zainab Zuhair Knaish Al Ghrabiu, Mohamed Kahloul

**Affiliations:** ^1^Department of Anesthesia and Intensive Care, Babil Teaching Hospital for Maternity and Children, Ministry of Health, Baghdad, Iraq; ^2^Department of Anesthesia and Intensive Care, University of Sousse, Faculty of Medicine Ibn AL Jazzar, Sousse, Tunisia; ^3^Department of Anesthesia and Intensive Care, Babil Health Directorate, Al Hilla General Teaching Hospital, Hillah, Iraq; ^4^Mathematics Department, University of Babylon, Hillah, Babylon, Iraq; ^5^Department of Anesthesia and Intensive Care, Sahloul Teaching Hospital, Faculty of Medicine of Sousse, University of Sousse, Sousse, Tunisia

**Keywords:** agitation, children, compliance, melatonin, orientation, surgery

## Abstract

**Background:** Following sedation or general anesthesia, emergent agitation (EA) presents as a sequence of abrupt, complicated psychomotor problems marked by perceptual abnormalities, delusions, and disorientation. Studies have proved that melatonin significantly decreases the incidence of postoperative agitation in children after anesthesia. The primary objective of this study was to compare the effectiveness of two doses of oral melatonin as a premedication for orientation score, induction compliance, and emergency agitation of children undergoing surgeries.

**Methods:** In this double-blinded randomized controlled trial, 126 children, aged 4–14, of either sex, with an ASA I or II, scheduled for elective surgery were randomly assigned to get either melatonin 0.4 mg/kg (Group M4) or melatonin 0.2 mg/kg (Group M2), with 63 kids in each group. All children have had the same anesthetic strategy. As a primary outcome, orientation score, induction compliance to intravenous induction anesthesia, and decreased emergency agitation were assessed.

**Results:** Both groups were comparable in terms of demographic characteristics and baseline data. Orientation scores were similar between the groups. Preoperatively, all patients were oriented in both time and place. The two groups had no statistically significant difference according to induction compliance distribution (*p*=0.065). There was a statistically significant difference in agitation behavior after 5, 10, and 15 min postoperatively in M 4, 2, and total participants (*p* < 0.001).

**Conclusion:** In pediatric surgical patients, the melatonin dosage does not affect children's compliance with induction but impacts their postoperative behavior by reducing the likelihood of agitation. Administering oral melatonin before surgery could potentially aid in managing postoperative delirium in children.

## 1. Introduction

Oral melatonin has increasingly gained attention as a premedication for elective pediatric surgeries. This research aimed to evaluate whether two doses of oral melatonin (7 mg) effectively improve the orientation score, induction compliance, and emergency agitation of children undergoing elective surgeries [1]. Oral melatonin syrup effectively reduces pediatric morning preanesthetic anxiety, increases preoperative surgical scores, promotes early recovery, and reduces emergence agitation, regardless of the time of surgery or anesthetic used [2, 3]. As one of the alternatives for preanesthetic management, the safe and appropriate oral dose of melatonin for pediatric preanesthetic medication still needs to be established [4].

Preanesthetic medication plays an important role in improving anesthetic outcomes among children. Different preanesthetic medications have been studied, starting with ketamine, midazolam, clonidine, and Melatonin, which are used orally [5].

Melatonin preparation can reduce preoperative anxiety and fear in adolescents. It is the drug of choice when assessing premedication and anesthetic induction [6]. Few studies exist concerning the effect of melatonin on three major anesthetic outcomes: anxiety based on the anxiety score, induction compliance, and emergency agitation based on the Pediatric Anesthesia Emergence Delirium Scale (PAEDS) in children [7].

There is a lack of available clinical data on the effects of melatonin in the premedication of the orientation score and emergency agitation in pediatric surgery [8]. The dose-response effects of melatonin as premedication on the orientation of children who had undergone general anesthesia and emergency agitation after the surgery have never been reported [9]. The primary objective of this study was to compare the effectiveness of two doses of oral melatonin as premedication on orientation score, induction compliance, and emergency agitation of children undergoing surgeries.

### 1.1. Patients and Methods

This was a double-blinded, randomized, controlled trial conducted in Babil Teaching Hospital for Maternity and Children, a tertiary children's hospital in Iraq, over a 16-month period (from November 2021 to February 2023).

After the ethics committee's approval (reference no. 59/10/2021) and parents' consent, one hundred twenty-six children aged 4–14, scheduled for an interventional elective surgery under general anesthesia and having an American Society of Anesthesiologists (ASA) physical status of I or II, were enroled.

Exclusion criteria were pediatric patients with physical status of ASA III or more, lung, heart, neurological, CNS disorder, diabetes, psychiatric illness, thyroid storm, drug allergy, liver disease, sleep disorders, intake of antipsychotics, or history of recent surgery. Patients who have already experienced sedation; those who have taken benzodiazepines, opioids, or any other sedatives in the past month; patients undergoing emergency surgery; or who declined to participate were also excluded from the study.

Before surgery, each patient was evaluated completely, including a history review, physical examination, and laboratory tests. The study eliminated patients who failed to satisfy the inclusion requirements.

Children were randomized into two groups (Group M2 and Group M4), including 63 patients each. They had oral premedication with melatonin 60 min before anesthesia, at 0.2 mg/kg (max 10 mg) in Group M2 and 0.4 mg/kg (max 10 mg) in Group M4. One milliliter of the used syrup was equal to 2.5 mg of melatonin.

### 1.2. Sample Size Calculation

The sample size was calculated with a given 95% confidence interval, a 0.5 standard deviation (anticipated variance), and a 5% margin of error, as follows.

In this equation *n* = [*Z*1−*a*/2+*Z*1−*B*/ES]^2^, *α* represents the chosen significance level, Z1−*α*/2 is the value from the standard normal distribution corresponding to 1−*α*/2, 1−*β* is the desired statistical power; *Z*1−*β* is the value from the standard normal distribution corresponding to 1−*β*; and ES represents the effect size.

The predicted sample size was raised to account for any expected dropouts (10%). The current study included 126 participants.

### 1.3. Randomization

An envelope method and a computer-generated random array were used to divide the included patients into two groups. Simple randomization occurred on a 1:1 ratio of melatonin 0.2 mg/kg (max 10) to the other melatonin dose of 0.4 mg/kg (max 10). Both doses of melatonin were taken as syrup in the same quantity and given to the child per kg. Each parent was assigned a private data form coded with the serial number. The surgical team that carried out the procedures comprised three pediatric surgeons. Researchers who did not participate directly in patient care prepared and administered the experimental drugs and involved surgeons, accountable anesthesiologists, and participating families who were unaware of the medication administration and group assignment.

All children were kept off oral intake on the day of operation for a minimum of 6 h for solids and 2 h for clear fluids. Premedication with melatonin was administered 60 min before the start of general anesthesia in both groups.

All patients have had general anesthesia. They were preoxygenated for 3 minutes. Ketamine 1–2 mg/kg and propofol 2–3 mg/kg induced anesthesia. Following induction, a laryngeal mask airway was inserted. 2% sevoflurane ensured anesthesia maintenance. The depth of anesthesia was controlled by varying the concentration of sevoflurane while keeping the oxygen flow rate at 2 L/min.

Orientation was assessed using an orientation score [10] (0 = none, 1 = orientation in either space or time, and 2 = orientation in both space and time).

Induction compliance among patients was assessed using the pediatric anesthesia behavior score (induction compliance) [11] as follows:

1. Happy, calm, and controlled. Compliant with induction
2. Sad, tearful, and withdrawn but compliant with induction
3. Mad loud vocal resistance (screaming or shouting)

The PAEDS was measured on admission to the PACU every 5 min and recorded at 5, 10, and 15 min, and the score was classified as follows: 1: awake and calm, cooperative; 2: crying, requires consoling; 3: irritable/restless, screaming, and inconsolable; and 4: combative, disoriented, and thrashing [12].

No supplemental analgesic agent was given, and a PAEDS was measured on admission to the PACU, every 5 min, and recorded during the postoperative period.

Children with an agitation score of 3 or 4 were classified as agitated. Although scoring agitation at 5-min intervals may not be ideal, in the busy milieu of our pediatric PACU, it was the most suitable and practical approach.

### 1.4. Statistical Analysis

Patients' data were collected and tabulated to prepare for statistical analysis using the SPSS programme. Data were presented as mean ± SD and percentage. A chi-square test was employed to compare observed outcomes with anticipated outcomes.

## 2. Results

### 2.1. Demographic Characteristics of the Studied Patients

The study included 126 children who underwent elective surgeries. Patients were randomly assigned to two groups: M4 (melatonin 0.4 mg/kg), which included 63 children, and M2 (melatonin 0.2 mg/kg), which included 63 other patients. The mean age of M4, M2, and total patients was 8.59 ± 2.89, 7.78 ± 3.18, and 8.18 ± 3.06, respectively, with no significant difference between the two groups (*p*=0.254). More than half of the patients in Groups M4 and M2 were males (69.8%, 58.7%, and 64.3%, respectively). The mean weights of patients in M4, M2, and total were 28.24 ± 11.06, 26.32 ± 11.96, and 27.28 ± 11.51 kg, respectively, with no statistically significant difference among groups (*p*=0.351) (Table 1).

### 2.2. Orientation Scale

Orientation scores were similar between the groups. Preoperatively, all patients were oriented in time and place (Table 2).

### 2.3. Induction Compliance

The M2 group had a higher number of sad, tearful patients (39.7%) and mad, loud vocal resistance (14.3%) than M4 patients (23.8% and 9.5%, respectively). However, according to the induction compliance distribution (*p*=0.065) (Table 3), there was no statistically significant difference between the two groups.

### 2.4. PAEDS

PAEDS score among the studied groups: After 5 min postoperatively, all patients in all groups were asleep. Ten minutes postoperatively, only one patient showed agitation and thrashing around in the MT 0.4 mg/kg dose group with a statistically significant difference (*p*=0.01). After 15 min, 20 children (31.7%) in the MT 0.2 mg/kg group showed an agitated response, which was higher than the MT 0.4 mg/kg group (9 patients [14.3%]). There was a statistically significant difference in agitation behavior after 5, 10, and 15 min postoperatively in M4, 2, and total participants (*p* < 0.001) (Table 4).

## 3. Discussion

Melatonin, synthesized by the pineal gland, is essential for sleep control through MT1 and MT2 receptors. Comprehensive studies have investigated its perioperative application for its sedative, anxiolytic, and analgesic effects. [13]. It has been reported to raise sedation levels without compromising orientation and causing preoperative anxiolysis. Children's preoperative anxiety is linked to a variety of postoperative outcomes, including eating disorders, bedwetting, longer recovery-phase suffering, and postoperative regressive behavioral abnormalities [14, 15].

There are a few trials on children receiving preoperative oral MT (0.2–0.5 mg/kg). One of these [16] has demonstrated that oral MT (0.5 mg/kg) is ineffective as a premedication in kids. The other people who have used oral MT (0.25 and 0.5 mg/kg) have shown encouraging results [17, 18].

The current study's demographic data (age, sex, and weight) were comparable in both groups. The mean age of M4, M2, and total patients was 8.59 ± 2.89, 7.78 ± 3.18, and 8.18 ± 3.06, respectively. The total male-to-female ratio was 2:1. In contrast, another study revealed that the ratio of men to women was about 1:1 across all groups, making them comparable and that the difference in weight across all groups was insignificant [19].

Several prior studies claimed that children who underwent sevoflurane anesthesia had a higher incidence of emerging agitation than those who underwent halothane anesthesia. However, some prospective studies assessing this claim found no significant difference between the two anesthetics [20–23]. Kuratani and Oi [20] revealed that in comparison to halothane, sevoflurane anesthesia frequently caused agitation upon awakening in pediatric patients. Their findings are in line with the current understanding among pediatric anesthesiologists.

The causes of emerging agitation include pain, pr-operative worry, the type of surgical operations, the patient's personality, too rapid waking, and the anesthetics used. The cause of emerging agitation cannot be attributed to one specific element [24]. Although pain is undoubtedly a substantial contributor to emergence agitation, screaming in response to pain needs to be differentiated from emergence agitation. However, it is occasionally impossible to tell them apart, particularly in younger children. It is commonly accepted that decreasing or eliminating pain after sevoflurane anesthesia lowers the likelihood of emerging agitation. Numerous investigations revealed that the use of regional blocks, opioids, and nonsteroidal anti-inflammatory medications reduces the likelihood of emerging agitation [21, 22, 24–26]. However, emerging agitation frequently happens even after good pain management or during nonpainful treatments. Weldon, Bell, and Craddock [21] revealed that when reliable postoperative pain management is provided with a caudal block, sevoflurane is linked to an early, temporary rise in the incidence of emerging agitation compared to halothane. In addition, comprehensive pain management does not ensure a peaceful awakening following sevoflurane if we take into account the possibility that anesthesia for almost pain-free procedural sedation can result in emerging disturbance [27]. In another study [28], even though esophagal dilatation is not painful, the patients in the placebo group were more agitated than the other patients.

Preoperative midazolam and melatonin were evaluated in two recent investigations in children [17, 29]. They reported that melatonin was superior to midazolam in reducing emerging delirium, the same as in the present trial. However, they also claimed that melatonin was just as powerful an antianxiety agent as midazolam taken orally. This latter discovery merits careful consideration. The validity of this earlier research may have been compromised by methodological issues, as stated in the introduction [17, 29]. The anxiolytic effects of melatonin in the earlier research could be explained by difficulties linked to the lack of adequate assessment methods and failure to account for the time of day the trial was conducted. A certified dose of melatonin and approved outcome instruments were used in the current investigation, a double-blind, randomized controlled trial.

Postoperatively, emerging delirium is more common in patients with higher preoperative anxiety levels [30]. Temperament and prehospital anxiety in a kid are risk variables for emergency delirium, which are challenging to manage in a research environment. A multimodal anxiolytic technique was integrated into the current study design to increase the likelihood of lowering the emergence of delirium.

According to a recent study, the scale ranges from 22.92 to 100, with a score of 30 or above signifying anxiety [31]. In all the groups, since preoperative anxiety levels were identical, the difference between preoperative melatonin and midazolam's effects on emergence delirium suggests that melatonin has a positive pharmacological effect on factors other than anxiolysis in preventing emergence delirium. It is known that neural remodeling of GABA receptors with a consequent decrease in binding sites is a possible mechanism of chronic posttraumatic delirium [32]. In addition, the current research suggests that the development of delirium may be influenced by activity on GABA receptors [33]. Melatonin boosts GABAergic transmission and raises GABA concentrations in the central nervous system, thereby enabling neural inhibition, which may explain the efficiency of melatonin in lowering emerging delirium [34].

The doses utilized in earlier studies of oral melatonin to avoid emerging delirium ranged from 0.05 to 0.5 mg kg [35]. Previous research on the effectiveness of melatonin in reducing the emerging delirium has revealed varying outcomes due to the different doses, with [17] showing a lower incidence [28], describing a comparable occurrence, and [36] reporting that melatonin treatment was associated with a greater frequency of emerging delirium than was midazolam. However, these trials did not use a multimodal strategy to reduce preoperative anxiety. Melatonin's impact on emerging delirium was considerably dose-dependent, with potential efficacy at doses of at least 0.2 mg kg, according to a meta-regression study [37]. In a survey by Kain et al., the authors stated that the dose of 0.3 mg kg establishes a balance between effectiveness and avoiding postoperative drowsiness [36].

There were no changes in orientation scores in the melatonin and placebo groups before or 60–90 min after giving premedication. Hence, the *p* value was not applicable. There was a difference in the midazolam group, which was not statistically significant (*p*=0.345). This showed that melatonin did not produce any change in orientation [14].

The MT 0.75 mg/kg group had the maximum number of patients with successful venepuncture compliance. The results were not statistically significant when MT 0.75 mg/kg was compared with midazolam and MT 0.5 mg/kg (*p*=0.6371 and *p*=0.1238, respectively) [38].

## 4. Limitations

This study had limitations, such as the absence of a placebo-controlled group and the number of participants, which could affect the generalizability of the findings. The chosen research methodology or design may have inherent limitations, such as potential biases or confounding variables that cannot be fully controlled, mainly when used in children. The research findings may be limited in generalizability to other populations and settings.

## 5. Conclusion

In pediatric surgical patients, the melatonin dosage does not affect children's compliance with induction but impacts their postoperative behavior by reducing the likelihood of agitation. Administering oral melatonin before surgery could potentially aid in managing postoperative agitation in children.

## Figures and Tables

**Table 1:** Demographic characteristics of the studied groups.

Variable	Parameter	M4 (*n* = 63)	M2 (*n* = 63)	Total (*n* = 126)	Test	*p* value
Age	Mean ± SD	8.59 ± 2.89	7.78 ± 3.18	8.18 ± 3.06	*χ* ^2^ = 2.74	0.254
	Range (min–max)	4–14	4–14	4–14		
	4–7 years	26 (41.3%)	33 (52.4%)	59 (46.8%)		
	> 7–10 years	16 (25.4%)	17 (27%)	33 (26.2%)		
	> 10 years	21 (33.3%)	13 (20.6%)	34 (27%)		

Sex, *n* (%)	Male	44 (69.8%)	37 (58.7%)	81 (64.3%)	*χ* ^2^ = 1.69	0.196
	Female	19 (30.2%)	26 (41.3%)	45 (35.7%)		

Weight	Mean ± SD	28.24 ± 11.06	26.32 ± 11.96	27.28 ± 11.51	0.876	0.351
	Range (min–max)	14–57	11–55	11–57		

*Note:p* value: the difference between M4 and M2. %, percentage; *χ*^2^, chi-square; *t*, student's *t*-test. *p* nonsignificant if > 0.05.

Abbreviations: Max, maximum; Min, minimum; *n*, number; SD, standard deviation.

**Table 2:** Orientation scale among the studied groups.

Variable	M4 (*n* = 63)	M2 (*n* = 63)	Total (*n* = 126)	Test	*p* value
None	0	0	0	—	—
Orientation in either time or place	0	0	0		
Orientation in both	63 (100%)	63 (100%)	126 (100%)		

*Note:p* value: the difference between M4 and M2. %, percentage; *χ*^2^, chi-square. *p* nonsignificant if > 0.05.

**Table 3:** Induction compliance score among the studied groups.

Variable	M4 (*n* = 63)	M2 (*n* = 63)	Total (*n* = 126)	Test	*p* value
Happy, calm, and controlled. Compliant with induction	42 (66.7%)	29 (46%)	71 (56.3%)	*χ* ^2^ = 5.48	0.065
Sad, tearful, and withdrawn but compliant with induction	15 (23.8%)	25 (39.7%)	40 (31.7%)		
Mad loud vocal resistance (screaming or shouting)	6 (9.5%)	9 (14.3%)	15 (11.9%)		

*Note:p* value: the difference between M4 and M2. %, percentage; *χ*^2^, chi-square. *p* nonsignificant if > 0.05.

**Table 4:** Emergency PAED score among the studied groups.

Variable	M4 (*n* = 63)	M2 (*n* = 63)	Total (*n* = 126)	*p* value
*5 min*				—
Asleep	63 (100%)	63 (100%)	126 (100%)	
Calm	0	0	0	
Crying but consolable	0	0	0	
Crying but inconsolable	0	0	0	
Agitated and thrashing around	0	0	0	

*10 min*				0.01⁣^∗^
Asleep	9 (14.3%)	0	9 (7.1%)	
Calm	51 (81%)	62 (98.4%)	113 (89.7%)	
Crying but consolable	2 (3.2%)	1 (1.6%)	3 (2.4%)	
Crying but inconsolable	0	0	0	
Agitated and thrashing around	1 (1.6%)	0	1 (0.8%)	

*15 min*				0.072
Asleep	3 (4.8%)	0	3 (2.4%)	
Calm	47 (74.6%)	41 (65.1%)	88 (69.8%)	
Crying but consolable	1 (1.6%)	1 (1.6%)	2 (1.6%)	
Crying but inconsolable	3 (4.8%)	1 (1.6%)	4 (3.2%)	
Agitated and thrashing around	9 (14.3%)	20 (31.7%)	29 (23%)	
*p* value	MC < 0.001⁣^∗^	MC < 0.001⁣^∗^	MC < 0.001⁣^∗^	

*Note:p* value: the difference between M4 and M2. %, percentage; *χ*^2^, chi-square. *p* nonsignificant if > 0.05.

Abbreviation: MC, Monte Carlo.

## Data Availability

The data supporting this study's findings are not publicly available due to privacy and confidentiality but are available from the corresponding author upon reasonable request.
